# Impact, challenges and limits of inpatient palliative care consultations – perspectives of requesting and conducting physicians

**DOI:** 10.1186/s12913-020-4936-x

**Published:** 2020-02-04

**Authors:** Anja Coym, Karin Oechsle, Alena Kanitz, Nora Puls, David Blum, Carsten Bokemeyer, Anneke Ullrich

**Affiliations:** 1 Palliative Care Unit, Department of Oncology, Hematology and BMT, University Medical Center Hamburg-Eppendorf, Martinistr. 52, 20246 Hamburg, Germany; 2 Competence Center Palliative Care, Department of Radiation Oncology, University Hospital Zürich, Zürich, Switzerland; 3 Department of Oncology, Hematology and BMT, University Medical Center Hamburg-Eppendorf, Martinistr. 52, Hamburg, Germany

**Keywords:** Palliative care, Inpatient palliative care consultations, Palliative care needs, Qualitative research

## Abstract

**Background:**

Inpatient palliative care consultation (IPCC) teams have been established to improve care for patients with specialist palliative care (PC) needs throughout all hospital departments. The objective is to explore physicians’ perceptions on the impact of IPCC, its triggers, challenges and limits, and their suggestions for future service improvements.

**Methods:**

A Qualitative study drawing on semi-structured interviews with 10 PC specialists of an IPCC team and nine IPCC requesting physicians from oncology and non-oncological departments of a university hospital. Analysis was performed using qualitative content analysis.

**Results:**

PC specialists and IPCC requesting physicians likewise considered organization of further care and symptom-burden as main reasons for IPCC requests. The main impact however was identified from both as improvement of patients’ (and their caregivers’) coping strategies and relief of the treating team. Mostly, PC specialists emphasized a reduction of symptom burden, and improvement of further care. Challenges in implementing IPCC were lack of time for both. PC specialists addressed requesting physicians’ skepticism towards PC. Barriers for realization of IPCC included structural aspects for both: limited time, staff capacities and setting. PC specialists saw problems in implementing recommendations like disagreement towards their suggestions. All interviewees considered education in PC a sensible approach for improvement.

**Conclusions:**

IPCC show various positive effects in supporting physicians and patients, but are also limited due to structural problems, lack of knowledge, insecurity, and skepticism by the requesting physicians. To overcome some of these challenges implementation of PC education programs for all physicians would be beneficial.

## Background

Early integration of palliative care (PC) has become increasingly accepted especially in cancer patients [1]. Even though the optimal time point and extent of PC is still in debate [2], there is little doubt of a general benefit [3]. It has shown to improve patients’ quality of life and symptom control [4], to reduce psychological distress for patients and family caregivers [5, 6], and to decrease health care costs due to for example less intensive care unit treatment [7]. It is anticipated that many patients facing an advanced, life-limiting disease would benefit from PC, and only a minority is provided with such service [8]. The demand for specialist PC in hospitals cannot be covered by PC units only [9, 10], and it concerns a large variety of chronic diseases – not only cancer [6, 11, 12]. Inpatient palliative care consultation (IPCC) teams have been established and shown a lowering of symptom burden in patients and their family caregivers [13–15].

The main reason for requesting IPCC is symptom management, especially pain management [9, 15], but also a demand for social and communicative support [16], including patient and family distress as well as help with decision making [17]. An increasing request for PC visits also seems to develop in departments like intensive care and cardiology, particularly in scenarios of prolonged intensive care treatment or repeated admissions. However, there is little research on how and when non-PC physicians see the indication for IPCC.

Existing literature indicates some challenges and limitations for IPCC: Utilization of IPCC seems to be dependent on information and education, since physicians and patients will not consider PC treatment without having knowledge about it and its availability [18]. Often it is believed that including IPCC is only possible for dying patients [3], or that it might be a signal for the patient that one has “given up on him” [19]. Thus, it appears that including IPCC is dependent on the individual physicians’ attitude. Very weak patients or patients close to death who cannot speak for themselves and express their needs and symptom burden are challenging and might have benefitted from an earlier integration of PC; not only for symptom control, but also for decision making [20–22]. In addition, insufficient IPCC staffing and logistical problems like short patient stays and difficulty in timing the consultation are challenging issues [23].

Despite these difficulties, IPCC has demonstrated positive effects in several areas of patient care: A multiprofessional palliative assessment covers more aspects than the ones addressed by the requesting physician [15, 22], and IPCC recommendations – pharmacological and non-pharmacological – can lead to less invasive measures which can be a relief for patients [24]. Prior studies reported pain relieve [21], but also improvement of nausea, depression, anxiety, insomnia and general well-being [5, 20]. Improvement of mental well-being after IPCC seems to be based, inter alia, on spiritual care and high quality communication with patients and their family caregivers [24, 25]. It leads to strengthened relationships and improved emotional stability especially concerning confrontation with death [4]. Additionally, IPCC has shown to enhance patients’ awareness of the disease including its prognosis [26].

However, PC in general is mostly accessible in high-income countries [27, 28], an issue addressed by the WHO, but still not covered satisfactory [29].

In Germany IPCC is quite a young field (cost covering since 2017) and therefore needs to grow and develop [30]. Especially since Europe has a longer tradition of providing PC through PC units, whereas it is the other way around in the United States of America [31].

In general, there are little data on utilization, barriers and effects of IPCC focusing on the views of PC specialists and requesting physicians. Previous studies have mostly explored perspectives of patients and PC specialists [20, 24, 32, 33]. Therefore, this qualitative study aims at exploring triggers leading to IPCC, the perceived impact, challenges, limits of IPCC and potential improvements from the perspectives of PC specialists of a multiprofessional IPCC-team and requesting physicians who have regularly requested support.

## Methods

### Study design

This multiperspective qualitative study was conducted in a maximum care hospital in Hamburg, Germany with 1700 beds and about 500,000 treated patients (in- and outpatients) per year. Almost all medical departments are represented except for e.g. geriatric medicine. Only a minority of physicians is educated in PC, even though critically ill patients – often without a curative intention - are treated in the different departments. Semi-structured interviews with PC specialists and regularly IPCC requesting physicians were conducted following an interview guide, which was developed based on literature and discussions within the multiprofessional PC research team. The ethics committee of the General Medical Council of Hamburg approved the study protocol (reference number 4981). Written informed consent was obtained from all study participants.

### Participants and setting

PC specialists were recruited from the local IPCC team. The hospital in this study provides this service since 2008, initially conducted by volunteer physicians only. Over the years, it expanded and holds a team with physicians and nurses since 2017; the requests have increased. It is available throughout the whole hospital and all departments via an online form in the electronic patient record. The PC department distributes brochures to inform about this service and pocket cards that help to identify PC needs. Physicians were eligible if having provided IPCC on a regular basis within the last 12 months. Requesting physicians were selected from the hospital’s departments of oncology, dermatology, internal medicine, gynecology and intensive care using purposive sampling (clinical experience and discipline). Senior PC specialists who are part of the multidisciplinary research team identified eligible participants, and the researcher invited them to participate. Eligibility criteria were provision of day-to-day care for inpatients with advanced, life-limiting diseases and having requested IPCC regularly within the last 12 months. In both groups, eligible physicians were recruited by email, and none of the invited physicians refused to participate or dropped out after giving consent for study participation. We intended to obtain views from PC specialists providing IPCC und users of IPCC with different experiences in both groups.

### Data collection

Following interviewer training and a pilot interview, face-to-face interviews were conducted by three female interviewers (AK, NP: medical students in their final year, AU: sociologist with working experience), who were neither involved in IPCC nor known by the interviewee. Interviews, with only interviewer and interviewee present, took place in a small private conference room on the PC unit. No repeat interviews were carried out. Interviews were conducted between January 2017 and May 2018. We created a semi-structured interview guide using open-ended questions. The interview guide (Additional files 1 and 2) was developed based on literature and the clinical experience of the research team. We conducted a complete pretest interview with each group, supervised by AU, to verify the need of modification, which was not the case, so that these two interviews were included in the sample. We enquired about issues leading to an IPCC request, perceived impacts, challenges and limits of IPCC, and suggestions for service improvement. Interviews were audio-recorded, transcribed verbatim and anonymized. Quotes displayed in the manuscript were translated by one of the authors (German native speaker fluent in English) and double-checked with other study members to evaluate the meaning. Data on participant characteristics and IPCC provision/request were collected during the interview.

### Data analysis

We performed qualitative content analysis of the interview transcripts using an inductive coding approach [34]. The software program MAXQDA [35] facilitated data management and coding. Text segments were assigned to subcategories, which were than categorized into main categories. This phase included constant comparison to verify and refine early subcategories until final categories were conceptualized. Thus, we iteratively established a coding framework, regularly discussed within the research team. Content of categories and subcategories were defined in interpersonal memos. Next, two investigators (NP and AC), supervised by AU, independently coded the transcripts. Even though we pursued a qualitative approach, we added a quantitative part to ponder relevance [36].

Transcripts were not returned to the interviewees for corrections or feedback.

We used the Consolidated Criteria for Reporting Qualitative Studies (COREQ) framework to report on the design, analysis, and results of our study where applicable [37].

## Results

### Sample and interview characteristics

In total, 10 PC specialists and 9 IPCC requesting physicians were interviewed. Interviewees’ characteristics are shown in Table 1. The interviews had an average duration of 45 min (range 33–51) for PC specialists and 40 min (range 27–53) for requesting physicians.

**Table 1:** Interviewee characteristics

	PC Specialist *n* = 10	Requesting physician *n* = 9
Gender		
Male	6	3
Female	4	6
Age in years		
< 30	0	5
30–40	7	3
41–50	2	0
> 50	1	1
Work experience in years		
< 1	0	2
1–5	0	2
5–10	7	4
> 10	3	1
PC specialists work experience in years		
< 1	3	n/a
1–5	5	n/a
5–10	2	n/a
Department		
ICU	n/a	2
Oncology	n/a	2
Gynecology	n/a	2
Dermatology	n/a	1
Nephrology	n/a	2
IPCC-requests in the last 12 Months		
3–6 times	n/a	1
7–10 times	n/a	3
11–20 times	n/a	4
> 20	n/a	1

*PC* Palliative care, *IPCC* Inpatient palliative care consultation, *ICU* Intensive care unit, *n/a* Not applicable

### Identified categories

We identified a number of topics leading to an IPCC request, its impact, challenges, limitations and suggestions for improvement. Details on categories are shown in Table 2 with a definition of its meaning and distribution of emphasis from the point of view of both groups; a quantitative element was captured as well.

**Table 2:** Results of identified categories and subcategories with physicians’ indications concerning the addressed aspects

Categories and Subcategories	Indications (*n* = 19)		Details/Explanation
	PC specialist (*n* = 10)	Requesting physician (*n* = 9)	
“Issues leading to an IPCC request”			
Physical symptom burden	+++	+++	Included different physical symptoms in pts
Patients’ quality of life	–	+	When the treating team assumed that quality of life could be improved by IPCC
Psychological distress	+++	++	Included pts’ and family caregivers distress
• Patients	++	+	
• Family caregivers	+	+	
Overstraining	+++	++	When family caregivers and the treating team were overwhelmed with dealing with pts
• Family caregivers	+	+	
• Treating team (health care professionals)	++	+/++^a^	
Organisation of further care	+++	+++	Support needed in organisation of Out of hospital care or transfer to PCU
Social-legal matters	+	–	Aspects that needed counselling on social-legal matters, e.g. advance directive
Decision-making	+	–	Support needed talking to pts. about decisions / the pts’ situation / medical reasonability
Change of therapeutic goal	+	+	Support needed in talking to pts. about therapeutic goals / to discuss medical reasonability
Limited staff resources	(+)	++	Pts in need of specialized PC are often time consuming and treating teams cannot meet the needs and therefore ask for support
“Barriers on regular wards concerning treatment of patients with PC needs”			
Connection of further outpatient palliative care	+	++	Treating teams are not in contact with outpatient palliative care services and lack knowledge on how to organize it
Coping	(+)	(+)	Regular wards have limited access to psychosocial support to assist pts. to deal with their situation
Lack of privacy (single rooms)	++	++	Regular wards usually have limited single rooms and little options for private conversations
Resources of the requesting team			Requesting physicians can be overwhelmed by the complexity of symptoms and psycho-social needs of pts., and not competent to treat these, also regular wards lack the preferable extent of multidisciplinarity
• Overstraining	++	+	
• No multidisciplinarity	++	(+)	
• Lack of knowledge	++	++	
• Lack of time	+++	++	
“Impact of IPCC”			
Transfer of knowledge to the requesting team	+	(+)	Through IPCC non-PC teams are educated in PC
Relief for the requesting team	++	++	Time consuming care and advice concerning palliative situations can be yield to the IPCC-team
Relief for family care givers	+	(+)	IPCC teams include family care givers in their treatment approach which helps them to get about the situation
Better patient coping	+	++	IPCC supports pts. in coping with the disease/palliative situation
Improvement of symptom burden	+++	+	Included different physical symptoms in pts
Improvement of further care (outside of the hospital)	++	(+)	IPCC improves out of hospital care like organisation of hospice care or other connection to further PC support
“Limitations for the IPCC-Team”			
Limited insight and treatment options in complex cases	++	++	IPCC offers only limited time to get to know pts. and their habits. IPCC is also limited in time to grasp the course of (often long-lasting) disease
Limited resources	+++	+++	Due to limited (IPCC-) staff they are limited in their offers
“Barriers concerning request, conduct and implementation of IPCC”			
Request			
Refusal of patients and family care givers	++	++	Pts/Family care givers refuse IPCC before having spoken to a IPCC-member
Fear of denigration	+	–	When requesting physicians fear of not having things done correctly and be showed up in front of colleagues
Overconfidence / Resistance	+++	(+)	When non-PC-physicians believe they know what’s best for the patient and don’t accept any other approaches and therefore do not request IPCC support
Lack of knowledge of the requesting team	+++	+	Without adequate knowledge the requesting team cannot identify situations or patients that/who would profit from PC.
Limited time	–	+	Limited time to fill out the request form or even to think about treating options in terms of PC
Assumption of missing benefit for the patient	–	+	The treating team feels that there is no benefit for pts. from additional PC treatment
No problems at all	–	+++	Meaning that no aspect prevents actions completely
Conduct			
No provision of an adequate setting	–	–	Regular wards have littler privacy, mostly no single rooms or meeting rooms with the option of speaking in private
Lack of preparation by the requesting team	++	–	When the IPCC team arrives, the treating team neglected to tell pts. about including IPCC or they have scheduled a treatment and pts. is therefore not available for consultation
Limited time	++	+	Limited time of the treating team so they are not open to discuss the situation and treatment options with the IPCC team
Limited time of the IPCC-Team	++	+	To complete all requests during a day there is limited time for pts
Patients refusal	–	(+)	Pts reject a consultation when actually meeting with the IPCC team
No problems at all	+	++	Meaning that no aspect prevents actions completely
Implementation of IPCC-suggestions			
Patients’ or family care givers’ refusal	(+)	(+)	After IPCC pts./family care givers reject the proposed approach
Insecurity, lack of knowledge	+++	(+)	The treating team feels uncomfortable with the proposed approach and therefore do not implement it, due to insecurity and a lack of knowledge
Resistance, Ignorance	++	+	The treating team does not believe in the proposed approach and that it would not be more helpful than their own treatment
Limited time	++	–	Due to the treating teams limited time they do not read IPCC suggestions properly and/or do not adjust the medication or treatment plan
No problems at all	(+)	++	Meaning that no aspect prevents actions completely

Number of interviews aspects were mentioned: - = none, (+) = one time, + = up to 1/3, ++ = up to 2/3, +++ = up to 3/3; ^a^Threshold region

*PC* Palliative care, *IPCC* Inpatient palliative care consultation, *pts* Patients, *PCU* Palliative care unit

### Issues leading to an IPCC request

An often mentioned trigger in both groups was physical symptom burden. A requesting physician associated IPCC support for symptom burden with team-based needs: *“(...) the team is overwhelmed with patients in extreme pain (...)“*(participant number A007, male, <30 years old, 0.75 years of working experience), and a PC specialist confirmed: *“(…*) *general wards are possibly overwhelmed by the needed time for patients and by the complexity of symptom control, psychosocial needs and the obligation of explaining that lifetime is limited (…*)” (participant number K004, female, 30–40 years old, 6 years of working experience, 1 year PC experience).

Another trigger mentioned in both groups was organization of further care like support in organizing home/hospice care or transferring patients to the PC unit. In the context of transfer, scarcity of resources was a frequently addressed issue. A PC specialist reported: *“(…*) *mostly transfer requests are due to pressure of the treating department to generate clinic space (…*)“ (participant number K003, male, 41-50 years old, 8.5 years of working experience, 8 years PC experience)*,* which was seen similarly by a requesting physician: *“(…*) *sadly the PC unit is mostly seen as a place to transfer dying patients to, to make room for new patients.”* (participant number A001, female, > 50 years old, > 10 years of working experience).

Decision-making was only spontaneously identified by a few PC specialist as a reason for IPCC support.

Some potential aspects were not identified as triggers for IPCC, but were addressed to other occupational groups, like spiritual and ethical questions or social-legal matters. Regarding the change of therapeutic goal, only a few interviewees thought of it as a reason to include IPCC.

Altogether, PC specialists identified more triggers leading to an IPCC request than requesting physicians.

### Perceived impact of IPCC

PC specialists and requesting physicians likewise saw a benefit for the treating team in terms of relief by backing up the team and giving advice. As said by one of the requesting physicians: *“we are very thankful for support from the IPCC with suggestions for further treatment (...) taking aspects into consideration we have not thought of. If there is no transfer option to the palliative care unit, one can always call and ask for help.”* (participant number A003, female, 30–40 years old, 6 years of working experience). Improvement of symptom management/control (mostly perceived as an impact by PC specialists) was reported not only leading to a relief for the patient but also the treating team, as mentioned by a PC specialist: *“we definitely reduce symptom burden and improve medical care. And I think the colleagues are thankful for our suggestions and support.”* (participant number K008, male, 30–40 years old, 9 years of working experience, 5 years PC experience). This was also supported by requesting physicians.

Additionally, both groups, but mostly requesting physicians, observed a positive impact on patient’s coping with the situation: *“after requesting IPCC, ( …*) *patients are less fearful concerning PC. They understand that PC does not mean they have to die right away (…*)” (participant number A005, female, < 30 years old, 1.5 years of working experience)*.* Relief of family caregivers was addressed, but less often.

Only one requesting physician and a few PC specialists mentioned improvement of further care (out-of-hospital with support of home care service and provision of aids) as an impact of IPCC.

### Challenges and limits of IPCC

Challenges of initiation and conduct, as well as implementation of IPCC recommendations were mostly mentioned by PC specialists.

PC specialists experienced lack of specialized knowledge of the treating team concerning palliative conditions. They also addressed overconfidence, as one specialist expressed: *“(…*) *colleagues who are unwilling to ask for palliative care support (…*) *perhaps because of a disproportionate self-esteem, thinking they can manage it by themselves.”* (participant number K001, female, 30–40 years old, 8 years of working experience, 4 years PC experience). Furthermore, limited time capacities were noted as a risk for not including IPCC: not taking it into consideration and not completing a request form.

Regarding the conduct PC specialists addressed that a proper introduction of PC was preferable before their consultation and not always done. Further, structural problems like missing adequate settings while also dealing with limited time capacities was challenging.

Several challenges of implementing IPCC recommendations were mentioned by PC specialists, whereas the majority of requesting physicians did not see any difficulties. Again, limited time capacities were addressed: PC specialists expressed that they find their recommendations not implemented when rechecking a case, assuming that is due to time difficulties, but they also identified situations where the treating team disagreed with the proposed approach.

### Suggestions for future service improvements

The interviewees were asked about necessary requirements for a successful co-treatment. PC specialists saw more information on the existence and accessibility of such a service as necessary. Requesting physicians felt that more personal contact in means of knowing the IPCC staff personally would be beneficial, as well as educational programs for them and their teams: *“(…*) *special knowledge is needed … that is something a “general physician” is not educated in (…*)” (participant number A007, male, < 30 years old, 0.75 years of working experience).

PC specialists suggested implementing systematic screening for PC need on all hospital wards as a method for earlier integration. Requesting physicians predominantly named more personnel resources for both groups to facilitate utilization of IPCC and earlier integration of PC, which was also mentioned by PC specialists.

## Discussion

This qualitative study explored triggers leading to IPCC, its impact, challenges and limits as well as suggestions for future service improvements. To gain insights into similarities and differences of perspectives of IPCC providers and users, we conducted semi-structured interviews with physicians from both groups.

Our findings show that there is a gap between the requesting physicians’ idea of why and how they might need support and the PC specialists’. In their narratives, PC specialists generally mentioned more triggers than the requesting physicians and identified more problems than addressed in the request, which indicates that education on PC for non-PC physicians could improve patient selection [15, 38].

The main reasons for asking for IPCC support seems to be physical symptom burden and help in organizing further care. The former is consistent with results of earlier studies throughout different countries [38–40], and is a very common connotation of PC. Organization of further care in terms of support in organizing home or hospice care or transferring patients to PC units was a frequently addressed issue and seems to be more present than in other studies. Explanations are pressure to shorten hospital stays on general wards [41], but also uncertainty and lack of knowledge on how to treat patients who have other needs than the “common” treatment the respective department focuses on.

Both groups identified overstraining of the treating team as a trigger, which gives the understanding that the requesting physicians acknowledge a lack of expertise in certain palliative situations, as it has been concluded in earlier studies [19].

Decision-making was narrated by a few PC specialists, while requesting physicians did not mentioned this trigger spontaneously. Studies show that decision-making is perceived as a relevant issue [42, 43], but possibly not as easily recognized or as explicitly addressed as physical symptom burden. Interestingly we also found, that changing the therapeutic goal was an aspect which does explicitly not trigger IPCC requests; probably because requesting physicians consider it their own duty. This is confirmed by other studies with physicians asking for support in end of life care discussions, but not in changing the therapeutic goal [6, 39, 42, 44].

Interestingly, the perception of impacts differed from the triggers of IPCC:

Improved symptom burden was identified as an impact, which is a finding well established in the literature [5, 20, 21, 45], however, in our study mostly mentioned by PC specialists and not requesting physicians, even though also the latter identified it as a trigger. An explanation might be that PC specialists focus more on symptoms than physicians without PC education.

Especially requesting physicians emphasized improved coping in patients and their family caregivers as a positive effect, even though only a few of them addressed it as a trigger. A positive impact on patient’s coping via an IPCC team has been described before [46]. Presumably, this leads to general relief for patients and physicians, which is crucial for improving patient care as a holistic approach [21]. It is surprising that less PC specialists than requesting physicians considered coping as an impact.

In our study, organizing further care was a main reason for requesting IPCC, but not so much seen as an impact. One explanation could be that the initial intention of the request was to transfer the patient to the PC unit oftenly, not as support for “out-of-hospital care”. This is usually addressed to the hospitals’ social service and therefore not a main aspect for IPCC. Earlier studies underscore that there is a need of clarification between the duties of a specialist IPCC social worker and the regular ward social worker [47].

PC specialists and requesting physicians perceived challenges and limits differently. PC specialists highlighted lack of knowledge of the treating team as an important aspect. This might cause a late request of PC support or that the IPCC-suggestions will be ignored [39]. PC specialists emphasized that a proper introduction of PC was preferable before visiting the patient and not always done. A previous study showed the importance of a valid explanation of the concept of PC beforehand [39], since without confronting patients with PC beforehand, they might still associate PC with oncoming death only [44]. However, there are of course patients who could benefit from PC, but cannot accept it, even after sufficient clarification [48].

Some of the above-mentioned challenges and limits were also due to limited time capacities. On the one hand, requesting physicians have to grasp the overall situation and think outside the box concerning their own field of expertise, which needs willingness and time. In addition, an understaffed team is more likely not to fill out a request form and not to take the suggested measures into account [23, 49]. On the other hand, PC specialists have limited time for each patient and also mandatory time-consuming documentation requirements. At the same time education is needed, which was addressed by requesting physicians and PC specialists, however literature indicates that non-PC-specialists are not as much in favor of receiving education than PC specialists in providing it [50].

### Strengths and limitations of the study

This study has several strengths. First, we included both perspectives: physicians conducting and physicians requesting IPCC. To our knowledge, this is the first study combining these views. Second, our sampling frame included physicians with different experience and from different medical disciplines. Third, data were analyzed, validated and discussed by a multidisciplinary research team including physicians and psychosocial researchers to ensure quality interpretation.

There are a number of limitations in our study. First, all interviewees were recruited from a single institution. Thus, transferability of our findings to other health care systems or different organized institutions is limited. Second, all interviewees in the requesting group were physicians known for including IPCC in their treatment, so obviously they have in general a positive attitude towards PC and we can assume that they are more likely to see reasons to include PC in their treating concept. It would be interesting to interview physicians who do not include IPCC in their daily practice for their reasons in a further study, including a focus on different specialties, since surgical disciplines are underrepresented. Interestingly there is data, which shows that surgical specialties experience less education in palliative care and therefore feel uncomfortable addressing it [51]. The interviews were conducted in a rather long period of time (19 interviews in 17 months); so that there could be a change of interviewees’ perception of IPCC additional to a maybe adjusted established practice in their daily work concerning PC patients. However, there were no changes concerning the structure or procedure of the IPCC-Team and no guideline changes.

Further, dynamics of mutual social desirability have to be taken into account: Medical students in their final year are familiar with medical terminology and hospital settings, which is an advantage for conducting interviews. At the same time, there might be a hierarchical gap when interviewing experienced physicians. We expect however to minimize these dynamics by interview training and supervision and the semi-structured interview approach. This endeavor of validating our findings included analysis and discussion of data by a multiprofessional research team.

## Conclusions

Findings of our study underscore the demand for PC support from a consultation team for various reasons. These include physical symptom control, organization of further care and coping/relief of patients and caregivers.

IPCC showed positive effects in supporting physicians including the whole treating team and patients concerning the mentioned aspects, but are also limited due to structural problems, lack of knowledge, insecurity, and skepticism by the requesting physicians. At the same time, more resources for both teams are needed to ensure continuity for patient treatment and further care. This fits with the made suggestions to improve IPCC services in terms of intensification of personal interaction between requesting and conducting physician, which is crucial for a joint treatment of patients. To achieve an improvement of PC management mandatory educational programs for health care professionals on all hospital wards could be sensible and help them to provide adequate care to their own patients. However, as mentioned before, staff resources in both groups are needed to provide educational offers and joint care of inpatients in need of PC.

To broaden the view on “non-users” it would be an interesting next step to include them in future studies on IPCC issues.

## Supplementary information

**Additional file 1.** Interview guide used for requesting physicians. A semi-structured interview guide using open-ended questions. The interview guide was developed based on literature and the clinical experience of the research team to obtain information adapted to the background of requesting physicians.

**Additional file 2.** Interview guide used for conducting physicians (PC specialists). A semi-structured interview guide using open-ended questions. The interview guide was developed based on literature and the clinical experience of the research team to obtain information adapted to the background of PC specialists.

## Data Availability

The authors have full control over the primary data. The data analyzed in this study are housed at the Palliative Care Unit, Department of Oncology, Hematology and BMT, University Medical Center Hamburg-Eppendorf, Martinistrasse 52, 20246 Hamburg, Germany. According to the ethical committee approval, this dataset is subject to ethical restrictions and local data protection regulations regarding qualitative raw data, since participant privacy could be compromised. Participants did not consent to have their full transcripts made available for third parties. All relevant data for the conclusions are presented in the manuscript.

## Abbreviations

COREQ: Consolidated Criteria for Reporting Qualitative Studies
IPCC: Inpatient palliative care consultation
PC: Palliative care
