# Supplementary material for: Impact, challenges and limits of inpatient palliative care consultations – perspectives of requesting and conducting physicians
Source: BMC Health Serv Res. 2020 Feb 4;20:86. doi: 10.1186/s12913-020-4936-x (PMC7001248; doi:10.1186/s12913-020-4936-x)
Supplement: Supplementary file 1 — Additional file 1. Interview guide used for requesting physicians. A semi-structured interview guide using open-ended questions. The interview guide was developed based on literature and the clinical experience of the research team to obtain information adapted to the background of requesting physicians. [file 12913_2020_4936_MOESM1_ESM.docx]

| I | What problems and needs lead to an IPCC request in a hospital setting (concerning patients with an incurable disease in non-PC specialist care)?  Prompts:   - on the part of the patients? - on the part of the family care givers? - on the part of the treating team? |
| --- | --- |
| II | What do you experience in terms of barriers in the regular treatment of patients with an incurable disease in a hospital setting (non-PC specialist care)? |
| III | In your opinion, what effect does IPCC support have concerning patients’ problems and needs during inpatient care (non-PC specialist care)? |
| IV | What kind of limitations do you perceive concerning IPCC co-treatment? |
| V | What problems and challenges concerning the request and conduct of an IPCC co-treatment do you experience? |
| VI | What problems and challenges concerning the implementation of IPCC recommendations do you experience? |
| VII | In your experience, are there any barriers that impede the request, conduct or implementation of recommendations completely? |
| VIII | In your opinion, what requirements must be met to successfully implement an IPCC co-treatment during inpatient care (non-PC specialist care)? |
| IX | In your opinion, what measures would help to improve non-PC specialist care (=general care) for patients with an incurable disease during inpatient treatment? |
| X | Is there anything we did not talk about which you think would be useful to know? |

**Additional file 1:** Interview guide used for requesting physicians.

IPCC: inpatient palliative care consultation; PC: palliative care
